# Between Vulnerability and Connection: Longitudinal Evidence on the Impact of Transformative Religious/Spiritual Experiences

**DOI:** 10.1002/smi.70110

**Published:** 2025-10-03

**Authors:** Zhuo Job Chen, Renae Wilkinson, Richard G. Cowden

**Affiliations:** ^1^ School of Nursing University of North Carolina at Charlotte Charlotte North Carolina USA; ^2^ Human Flourishing Program Institute for Quantitative Social Science Harvard University Cambridge Massachusetts USA; ^3^ Department of Epidemiology Harvard T.H. Chan School of Public Health Boston Massachusetts USA

**Keywords:** add health dataset, connectedness, prosocial, transformative religious/spiritual experiences, volunteering

## Abstract

Transformative religious/spiritual experiences (RSE) represent a subset of extraordinary experiences that are both self‐destabilizing and relational in nature. This double‐edged quality positions transformative RSE as both a potential source of psychological vulnerability and a catalyst for enhanced social connectedness. This study investigates the antecedents and outcomes of transformative RSE using a nationally representative longitudinal sample of 10,529 young adults from the National Longitudinal Study of Adolescent to Adult Health T0 (1994–1995), T1 (2001–2002), and T2 (2008). We examined associations of reporting a transformative RSE at T1 (late adolescence) on a broad range of physical, mental, behavioural, and social health and wellbeing indicators assessed at T2 (early adulthood). Primary analyses controlled for an extensive set of covariates assessed at T0 (early adolescence), with sensitivity analyses employing both liberal (T0 sociodemographic characteristics only) and conservative (contemporaneous covariates taken from T1) adjustment strategies. Antecedents (T0 correlates) of transformative RSE included adverse childhood environments, negative parental dynamics, and heightened religious involvement. Consistent T2 outcomes of transformative RSE involved some markers of mental and social vulnerability (i.e., PTSD diagnosis and loneliness), as well as increased prosocial engagement (i.e., volunteering and voting). These findings support the theorized double‐edged sword effect of transformative RSE and suggest the potential role of meaning‐making and integration in shaping long‐term psychological and social outcomes.

## 1 Introduction

‘Did you ever have a religious or spiritual experience that changed your life?’ When asked in the 2004 U.S. General Social Survey, over 35% of respondents reported having had such an experience (Smith 2006). A comparable item was asked in a national poll by Gallup (2003), in which 41% of Americans endorsed the statement, ‘I have had a profound religious experience or awakening that changed the direction of my life.’ Despite the relatively high prevalence of such transformative religious/spiritual experiences (RSE), and substantial research linking religious/spiritual involvement (e.g., service attendance) with better health outcomes and lower stress (for a scoping review, see Davis et al. in press), longitudinal evidence on the lasting health and stress‐related consequences of transformative RSE remains limited. Addressing this gap, the present study provides empirical evidence on the longitudinal associations between transformative RSE and a wide array of health, stress, and wellbeing outcomes, drawing on data from a nationally representative U.S. cohort tracked across three waves over a 14‐year span, from adolescence into adulthood.

### 1.1 Double‐Edged Sword Effect of Transformative RSE

The opening question touches on a broad spectrum of extraordinary experiences that both transcend perceived reality—appraised as religious or spiritual—and transform the individual. James (1902) was among the first to study such experiences empirically, summarizing his findings in the classic *The Varieties of Religious Experience*. Were the book written today, it might well be titled *The Varieties of Spiritual Experience* (Yaden and Newberg 2022), reflecting the growing number of individuals who more readily identify as spiritual than religious (Chen et al. 2023). Depending on definitional boundaries, transformative RSE may be situated within broader constructs like self‐transcendence (Yaden et al. 2017) or mysticism (Chen 2024). Regardless of the terminology, these experiences are too numerous and heterogeneous to be fully catalogued (White 1997), calling for analytical focus on the experiential features that are shared across diverse reports (Taves and Barlev 2023). As one example of this, a hierarchical model of mysticism has been put forward to organize different forms of transformative RSE into a single schema (see Chen 2024).

Regardless of how one might conceptually organize transformative RSE experiences, a central feature of them is that the ordinary sense of self is often disrupted or completely lost—but that is only one half of the story. The other half, which completes the transformation, is that the lost self is retrieved in a greater unity (Hood 2002). This dual nature of transformative RSE is recognized by Yaden et al. (2017) as ‘transient mental states of decreased self‐salience and increased feelings of connectedness’ (p. 143). Thus, transformative RSE can be a double‐edged sword. On one hand, they may imply a weakened sense of self—destabilized to create space for growth—but may also lead to depersonalization and a loss of personal boundaries. On the other hand, they involve a relational component, fostering a sense of connectedness with something beyond the self, often encompassing other people, the environment, or a larger cosmic context.

### 1.2 Weakened Self and Vulnerability

Transformative RSE may lie along a pathway of psychological vulnerability. These experiences are often preceded by periods of intense self‐examination, distress, or mental health challenges, as suggested in numerous qualitative studies (Garcia‐Romeu et al. 2015). In a qualitative study of 161 individuals who reported having temporary transformative experiences, 23% indicated that these were triggered by psychological turmoil or trauma—such as alcoholism, bereavement, serious illness, disability, or depression (Taylor 2012). Similarly, data from the 2004 General Social Survey found that the leading causes of spiritual or religious change were personal illness or accident, or the death or illness of someone close (Smith 2006; Wilkinson 2018). An analysis of 82 diverse RSE reports from the Hardy Database also suggests a common trajectory: an intense state of distress is followed by a sudden and often dramatic shift toward a more positive cognitive‐emotional state. This trajectory has been noted to resemble those of certain psychedelic and psychotic‐like experiences (Brouwer et al. 2025).

Not only are transformative RSE frequently preceded by vulnerable psychological states, but they may also be accompanied by challenging emotional content. For example, college students describe their spiritual transformation experiences as marked by uncertainty, obstacles, emotional turmoil, and negative emotions such as guilt, pain, sadness, and worry (Cohen et al. 2010). Even in the aftermath of such experiences, individuals often face prolonged and difficult integration processes. The very nature of these inner spiritual struggles can leave one susceptible to a deep sense of insecurity and confusion (Brook 2021).

Transformative experiences may become detrimental when they cannot be cognitively assimilated or meaningfully integrated. A review of 157 empirical studies emphasized that appraisal processes—how individuals interpret and make sense of the experience—play a fundamental role in shaping mental health outcomes (Maraldi et al. 2024). Without a coherent meaning‐making system, individuals may find it difficult to integrate these experiences into their worldview, which can have negative psychological consequences. Qualitative interviews with atheists who reported spontaneous RSE—anomalous given their worldview—found that those who struggled to interpret the experience and resolve lingering doubts reported deteriorating mental health (Van der Tempel and Moodley 2020).

Another contributing factor to the potential harm of transformative RSE is the phenomenon of weakened self. Experimental research has shown that recalling spiritual experiences can elicit a ‘small self’ perspective (Preston and Shin 2017). While this sense of diminished ego can be beneficial under certain circumstances, it may also mirror symptoms of depersonalization (DP) and derealization (DR)—psychological states characterized by a disturbing sense of detachment from the self or the surrounding environment. Prospective clinical studies have identified DP/DR symptoms as significant prognostic indicators in the course of depression and other health outcomes (Michal et al. 2024). Thus, the negative psychological effects of transformative RSE may, in part, be attributable to their depersonalizing impact.

### 1.3 Enhanced Connectedness and Meaning

However, a weakened sense of self may create space for connection to something greater, through which individuals often find deeper meaning in life (Wong et al. 2022). Theoretical models have proposed that transformative RSE may serve as a bridge between trauma and post‐traumatic growth. For instance, the Vulnerability‐Wellbeing Model posits that heightened vulnerability—such as crises involving health or mortality awareness—can naturally give rise to transformative RSE, which in turn mediates the relationship between vulnerability and wellbeing (Reed 2023).

Transformative RSE often lead to enhanced meaning‐making and a strengthened desire to cultivate connectedness in everyday life. This shift may manifest in improved interpersonal functioning and lifestyle decisions, as evidenced by qualitative interviews with both atheist (Van der Tempel and Moodley 2020) and religious individuals (Garcia‐Romeu et al. 2015). Data from the General Social Survey suggest that 17% of Americans who had undergone such experiences reported becoming better people by improving their character or behaviour—for example, by becoming more compassionate and selfless (Smith 2006). College student samples have similarly described major changes following RSE, citing greater insight into the self and the world, and the emergence of a renewed sense of purpose (Cohen et al. 2010).

The strongest empirical support comes from randomized clinical trials involving psychedelics. In studies using psilocybin to treat depression and addiction, participants frequently report experiences of ego loss and unity—phenomenologically similar to the transformative RSE discussed here. These experiences, compared to control conditions, significantly predicted increases in altruism, enhanced social connectedness, and heightened meaning, as assessed by both self‐report and third‐party ratings 2 months after the session (Griffiths et al. 2006). Moreover, the intensity of these experiences on session days was predictive of therapeutic outcomes, including reduced depression and anxiety (Griffiths et al. 2016). Related phenomena such as near‐death experiences (NDEs) have also been reported as profoundly transformative. An 8‐year longitudinal study involving cardiac arrest survivors in the Netherlands found that the 35 individuals who had NDEs—compared to the 39 non‐NDE patients—reported greater meaning in life, improved family involvement, enhanced acceptance of others, and a deeper sense of purpose (Van Lommel et al. 2001). These differences remained robust even after 8 years.

Nonetheless, most correlational studies have not found consistent positive associations between transformative RSE and wellbeing. For instance, using lifetime measures of mystical experiences centred on ego loss and unity, Francis and Robbins (2014) reported no significant associations between mystical experience and dimensions of psychoticism and neuroticism among adolescents in England and Wales, including 203 Muslims, 477 Christians, and 378 religiously unaffiliated individuals. Furthermore, longitudinal investigations remain scarce. In a 4‐year panel study of 1034 German and American adults, Chen and Streib (2022) similarly found little evidence of meaningful associations between mystical experiences and psychological wellbeing over time.

### 1.4 Current Study

Despite the common reports of transformative RSE, few studies have examined their long‐term implications for health and wellbeing within a longitudinal framework in larger populations (Maraldi et al. 2024). While theoretical models have emphasized the double‐edged nature of these experiences—both weakening the self and heightening the need for meaningful connection—empirical investigations rarely include relevant outcomes to test these dual effects. The present study sought to rigorously examine the longitudinal associations between transformative RSE and a broad range of indicators reflecting physical, mental, behavioural, and social dimensions of health and wellbeing, using a preexisting nationally representative dataset. Given the secondary nature of the data, we aimed to leverage the available variables both to explore novel empirical relationships and provide some insight into key theoretical propositions. Given the potential double‐edged nature of transformative RSE, we anticipated that reports of transformative RSE would be associated with poorer health outcomes (e.g., post‐traumatic symptoms) as indicators of vulnerability, and with higher levels of social engagement (e.g., volunteering) as behavioural expressions of enhanced need for connectedness.

## 2 Method

### 2.1 Study Sample

The analytic sample included *n* = 10,529 participants from the National Longitudinal Study of Adolescent to Adult Health (Add Health). Add Health is a prospective, nationally representative study that follows adolescents into adulthood (Harris 2013). Respondents were drawn from randomly selected schools across the United States, stratified by urban, suburban, and rural settings. In Wave I, students completed an in‐school survey (*N* = 90,118), after which a consenting subset of adolescents in grades 7–12 participated in in‐depth, in‐home interviews. During this wave, data were also collected from a residential parent (typically the mother) through a parent questionnaire, which included information on their current partner (usually the father). Subsequent waves of data collection followed this in‐home sample. Further details on the Add Health study are available on the study website (https://addhealth.cpc.unc.edu/).

This study used data from three waves: T0 (Wave I: 1994–1995, early adolescence), T1 (Wave III: 2001–2002, late adolescence), and T2 (Wave IV: 2008, early adulthood). We restricted the sample to participants who completed T1—the wave in which transformative RSE was assessed—and who also had valid sampling weights at T2 to account for the study's complex sampling design and attrition (*n* = 12,183). Although transformative RSE was assessed at T1, 1613 participants reported that their experience had occurred prior to their T0 interview. To reduce potential bias from T0 covariates acting as mediators or colliders, we made an a priori decision to exclude these participants. The final analytic sample therefore consisted of *n* = 10,529 participants who reported having a transformative RSE between T0 and T1.

### 2.2 Measures

#### 2.2.1 Exposure

In T1, participants were asked: ‘Did you ever have a religious or spiritual experience that changed your life?’ (*transformative RSE*). Response options were binary: 0 = no, 1 = yes. Participants also completed a follow‐up question that asked, ‘About how old were you the first time this happened?’. We used this variable to exclude participants if their experience occurred before T0.

#### 2.2.2 Outcomes

Consistent with previous studies, we assessed 38 outcomes that aligned with a multidimensional conception of health and wellbeing (Wilkinson et al. 2023; Long et al. 2024). The outcomes included: *physical health* (number of diagnosed physical health conditions, cancer, high cholesterol, hypertension, diabetes, asthma, migraines, allostatic load, overweight/obesity, functional limitations, cognition, and self‐rated health), *behavioural health* (sleep disturbance, physical inactivity, cigarette smoking, binge drinking, marijuana use, non‐medical prescription use, illicit drug use, history of sexually transmitted infections [STIs], preventative health care use), *mental health* and wellbeing (depressive symptoms, depressive disorder diagnosis, anxiety disorder diagnosis, posttraumatic stress disorder [PTSD] diagnosis, attention deficit disorder or attention‐deficit/hyperactivity disorder [ADD/ADHD] diagnosis, suicidal ideation, perceived stress, happiness, job satisfaction, optimism, sense of control), *social wellbeing and engagement* (loneliness, romantic relationship quality, satisfaction with parenting, relationship quality with parent, volunteering, voting). Further information about how each of these variables were assessed is available in Supporting Information S1: Text S1.

#### 2.2.3 Covariates

We included covariates based on data that were available (either through self‐report or parent‐report) and aligned with the application of the disjunctive cause criterion for confounder selection (VanderWeele 2019). Covariates were assessed at the wave prior to the exposure, reducing the likelihood that they function as mediators or colliders. These covariates included: sociodemographic and family background (e.g., age, sex, race/ethnicity, geographic region, household income), parental health and relationship (e.g., parent has obesity, smoker in household, childhood maltreatment by parents); psychosocial and academic factors (e.g., depressive symptoms, life expectancy, delinquency); health status and behaviour (e.g., somatic symptoms, suicidal ideation, cigarette smoking); and religious/spiritual practice (e.g., mother religious service attendance, religious service attendance, frequency of prayer). For additional details on the full set of covariates, see Table 1 and the note in Table 2.

**TABLE 1:** T0 characteristics of participants by transformative RSE status at T1 (*n* = 10,529).

Characteristics at T0	Had a transformative RSE at T1		
	No (*n* = 8731)	Yes (*n* = 1798)	*p*‐value
Sociodemographic and family background			
Age (range: 11–21), *M* (SD)	15.38 (1.81)	15.08 (1.77)	< 0.001
Female, %	50.22	52.89	0.001
Race/ethnicity, %			< 0.001
White	68.23	57.50	
Black	13.02	23.19	
Hispanic	11.89	11.83	
Asian	3.64	3.37	
Other	3.21	4.11	
Born in the U.S., %	94.94	94.89	0.091
Geographic region, %			< 0.001
Northwest	16.68	16.80	
Midwest	34.08	28.71	
South	35.05	44.22	
West	14.19	10.27	
Two‐parent household, %	70.33	65.08	0.011
Number of siblings (range: 0–12), *M* (SD)	1.39 (1.18)	1.40 (1.26)	0.357
Household income, %			0.047
1st Quintile	19.52	20.55	
2nd Quintile	19.28	21.67	
3rd Quintile	20.53	19.28	
4th Quintile	18.80	18.86	
5th Quintile	21.87	19.63	
Household welfare receipt, %	20.80	24.78	0.001
Has health insurance, %	88.25	90.73	0.097
Mother age (range: 23–81), *M* (SD)	40.97 (5.54)	41.06 (5.74)	0.950
Mother race/ethnicity, %			< 0.001
White	73.24	62.68	
Black	11.07	20.87	
Hispanic	10.08	11.47	
Asian	3.01	2.69	
Other	2.59	2.30	
Parents born in the U.S., %	84.01	84.06	0.001
Parental education, %			0.145
Less than high school	10.53	11.65	
High school equivalency	29.56	26.72	
Some college	26.27	26.19	
College degree or higher	33.63	35.44	
Mother employed full‐time, %	56.73	57.47	0.192
Parental health and relationship			
Mother self‐rated health (range: 1–5), *M* (SD)	3.64 (1.02)	3.61 (1.01)	0.155
Mother happy, %	88.95	87.53	0.437
Parent has a disability, %	13.92	12.50	0.990
Parent has obesity, %	22.98	27.46	0.023
Parent has alcoholism, %	17.32	18.51	0.745
Smoker in household, %	48.31	45.92	0.012
Childhood maltreatment by parents, %	19.04	21.39	0.002
Parental control (range: 0–7), mean (SD)	1.83 (1.58)	1.94 (1.62)	0.001
Parental relationship quality (range: 1–5), *M* (SD)	4.59 (0.71)	4.65 (0.68)	0.147
Psychosocial and academic factors			
Mental health condition diagnosis, %	5.42	5.54	0.139
Depressive symptoms (range: 0–3), *M* (SD)	1.63 (0.46)	1.68 (0.48)	< 0.001
Happiness (range: 0–3), mean (SD)	3.13 (0.80)	3.11 (0.80)	0.795
Self‐esteem (range: 1–5), mean (SD)	4.11 (0.59)	4.10 (0.61)	0.518
Perceived survival expectation (range: 1–5), mean (SD)	4.40 (0.67)	4.34 (0.69)	0.017
Has romantic partner, %	34.57	34.76	0.266
Has a learning disability, %	13.64	12.89	0.623
School connectedness (range: 1–5), *M* (SD)	3.70 (0.75)	3.72 (0.74)	0.561
Neighbourhood social cohesion (range: 0–5), *M* (SD)	3.85 (1.29)	3.84 (1.29)	0.414
PPVT (range: 13–146), *M* (SD)	101.69 (14.30)	100.87 (15.19)	< 0.001
GPA (range: 1–4), *M* (SD)	2.80 (0.78)	2.83 (0.75)	0.340
Delinquency (range: 0–15), *M* (SD)	2.76 (2.74)	2.98 (2.84)	0.043
Health status and behaviour			
Somatic symptoms (range: 0–4), *M* (SD)	1.76 (0.41)	1.80 (0.43)	< 0.001
Pubertal development (range: −10.23–9.59), *M* (SD)	0.13 (2.13)	0.05 (2.16)	0.921
Physical health condition diagnosis, %	22.80	23.70	0.082
Overweight/obesity, %	22.16	23.26	0.519
Functional limitations, %	0.70	0.40	0.583
Self‐rated health (range: 1–5), *M* (SD)	3.87 (0.91)	3.85 (0.90)	0.669
Suicidal ideation, %	13.45	14.87	0.027
Sleep disturbance, %	24.08	25.63	0.208
Physical inactivity, %	5.01	5.63	0.442
Cigarette smoking, %	18.45	16.17	0.003
Binge drinking, %	6.69	6.48	0.098
Marijuana use, %	15.72	15.61	0.345
Illicit drug use, %	12.14	12.30	0.838
History of STIs, %	2.57	3.53	0.176
Preventative health care use, %	64.67	66.91	0.212
Religious/spiritual practice			
Mother religious service attendance, %			< 0.001
Never or seldom	23.05	15.18	
Less than once a week	44.87	40.86	
At least once a week	32.08	43.96	
Religious service attendance, %			< 0.001
Never	28.74	20.90	
Less than once a week	38.85	35.04	
At least once a week	32.40	44.06	
Prayer, %			< 0.001
Never	24.34	15.34	
Once a week or less	41.42	35.76	
At least once a day	34.24	48.90	

*Note:* Table is based on non‐imputed data. *p*‐values come from chi‐squared or analysis of variance tests. Cumulative percentages for categorical variables may not add up to 100% due to rounding.

**TABLE 2:** Associations between reporting a transformative RSE at T1 and subsequent health and wellbeing in adulthood at T2, controlling for characteristics measured at T0 (*n* = 10,529).

Outcomes assessed at T2	*β* [95% CI]	RR/OR [95% CI]	*p*‐value	*E*‐value [limit]
Physical health				
Number of diagnosed conditions	0.03 [−0.04, 0.09]		0.446	1.18 [1.00]
Cancer		0.96 [0.52, 1.78]	0.894	1.25 [1.00]
High cholesterol		1.29 [0.98, 1.70]	0.068	1.91 [1.00]
Hypertension		0.99 [0.82, 1.20]	0.956	1.08 [1.00]
Diabetes		**1.50 [1.03, 2.19]**	0.035	2.37 [1.20]
Asthma		1.02 [0.88, 1.19]	0.794	1.16 [1.00]
Migraines		0.86 [0.73, 1.02]	0.078	1.58 [1.00]
Allostatic load	0.03 [−0.03, 0.10]		0.348	1.20 [1.00]
Overweight/obesity		1.01 [0.96, 1.06]	0.674	1.11 [1.00]
Functional limitations		**1.32 [1.04, 1.68]**	0.022	1.98 [1.25]
Cognition	0.02 [−0.04, 0.08]		0.510	1.16 [1.00]
Self‐rated health	**−0.08 [−0.14, −0.01]**		0.035	1.35 [1.08]
Behavioural health				
Sleep disturbance		1.02 [0.95, 1.09]	0.605	1.15 [1.00]
Physical inactivity		0.95 [0.80, 1.13]	0.579	1.28 [1.00]
Cigarette smoking		0.99 [0.89, 1.10]	0.856	1.11 [1.00]
Binge drinking		0.90 [0.74, 1.10]	0.295	1.46 [1.00]
Marijuana use		1.09 [0.93, 1.28]	0.294	1.40 [1.00]
Prescription drug misuse		**1.15 [1.01, 1.31]**	0.041	1.56 [1.08]
Illicit drug use		1.10 [0.99, 1.22]	0.089	1.42 [1.00]
History of STIs		0.99 [0.81, 1.20]	0.911	1.12 [1.00]
Preventative health care use		0.97 [0.92, 1.02]	0.199	1.22 [1.00]
Mental health and wellbeing				
Depressive symptoms	**0.06 [0.00, 0.12]**		0.044	1.30 [1.00]
Depression diagnosis		1.05 [0.90, 1.23]	0.525	1.28 [1.00]
Anxiety diagnosis		1.15 [0.98, 1.36]	0.084	1.58 [1.00]
PTSD diagnosis		**1.54 [1.09, 2.19]**	0.015	2.46 [1.40]
ADD/ADHD diagnosis		1.10 [0.72, 1.67]	0.668	1.42 [1.00]
Suicidal ideation		1.31 [1.00, 1.72]	0.051	1.95 [1.00]
Perceived stress	0.00 [−0.06, 0.07]		0.870	1.07 [1.00]
Happiness	−0.05 [−0.12, 0.02]		0.162	1.27 [1.00]
Job satisfaction	−0.00 [−0.08, 0.07]		0.933	1.06 [1.00]
Optimism	0.05 [−0.02, 0.12]		0.192	1.26 [1.00]
Sense of control	−0.03 [−0.09, 0.04]		0.370	1.19 [1.00]
Social wellbeing and engagement				
Loneliness	**0.13 [0.06, 0.19]**		< 0.001	1.49 [1.31]
Romantic relationship quality	−0.04 [−0.10, 0.03]		0.256	1.22 [1.00]
Satisfaction with parenting^	−0.03 [−0.12, 0.07]		0.550	1.19 [1.00]
Relationship quality with parent	0.02 [−0.06, 0.10]		0.691	1.14 [1.00]
Voting		**1.18 [1.09, 1.27]**	< 0.001	1.63 [1.41]
Volunteering		**1.21 [1.12, 1.30]**	< 0.001	1.71 [1.48]

*Note:* Each outcome is a separate regression model, and all models control for covariates assessed at T0: *sociodemographic and family background* (age, sex, race/ethnicity, nativity status, geographic region, family structure, number of siblings, household income, household welfare receipt, insurance status, mother age, mother race/ethnicity, parent nativity, parental education, mother employment status), *parental health and relationship* (mother health status, mother happiness, parent has a disability, parent has obesity, parent has alcoholism, smoker in household, childhood maltreatment by parents, parental control, relationship quality with a parent); *psychosocial and academic factors* (mental health condition diagnosis, depressive symptoms, happiness, self‐esteem, life expectancy, romantic relationship status, has a learning disability, school connectedness, neighbourhood social cohesion, PPVT, GPA, delinquency); *health status and behaviour* (somatic symptoms, pubertal development, physical health condition diagnosis, overweight/obesity, functional limitations, self‐rated health, suicidal ideation, sleep disturbance, physical inactivity, cigarette smoking, binge drinking, marijuana use, illicit drug use, history of STIs, preventative health care use); and *religious/spiritual practice* (mother religious service attendance, religious service attendance, frequency of prayer). ^Analysis for this outcome was restricted to participants who reported having at least one child at T2 (*n* = 5172). Bold values are statistically significant at *p* < 0.05.

Abbreviations: CI, confidence interval; OR, odds ratio; RR, risk ratio.

### 2.3 Data Analysis

All analyses were conducted using Stata 17.0. We first examined the characteristics of participants at T0 as a function of those who reported (vs. did not report) a transformative RSE at T1. This analysis, based on available data, was descriptive in nature for both groups. Chi‐squared tests were used for categorical variables, and analysis of variance was applied for continuous variables to assess group differences.

The primary analysis was conducted using imputed datasets. We followed standard procedures to impute missing data for covariates and outcomes using multiple imputation by chained equations. Five imputed datasets were generated to reduce sampling variability in the imputation process, and results were combined using Rubin's rule (Li et al. 2015). Following an outcome‐wide analytic framework (VanderWeele et al. 2020), we examined the longitudinal associations between transformative RSE at T1 and outcomes at T2, controlling for T0 covariates (including prior values of available outcomes assessed at T0). A separate regression model was estimated for each of the 38 outcomes, based on the nature of the outcome variable. For binary outcomes with prevalence < 10%, we used logistic regression, reporting odds ratios (OR). For binary outcomes with prevalence ≥ 10%, we used generalized linear models with a log link for Poisson distribution, reporting risk ratios (RR). For continuous outcomes, we used linear regression with standardized outcomes (mean = 0, standard deviation = 1), reporting betas interpreted as standard deviation change in the outcome.

We conducted several sensitivity analyses. First, we ran models with a reduced set of T0 covariates (i.e., sociodemographic characteristics only) that are traditionally included in the social sciences to assess whether the inclusion of a large number of covariates influenced results. Second, we ran models adjusting for contemporaneous covariates assessed at T1, including prior values of available outcomes, to examine the potential impact of covariate timing. While multiple imputation is known to reduce bias and improve statistical power, it relies on the assumption that data are missing at random (Enders 2022). We therefore reran the primary analysis using complete case analysis with pairwise deletion for missing values.

Given the 38 regression models estimated, we focused primarily on results with a *p*‐value less than 0.0013, applying a conservative Bonferroni correction to account for multiple testing (*p* = 0.05/38). In addition to reporting regression coefficients and 95% confidence intervals, we calculated *E*‐values for each effect and for the limit of the confidence interval closest to the null. Following the approach by VanderWeele and Ding (2017), this sensitivity analysis quantifies the minimum strength of association that an unmeasured confounder would need to have with both the exposure and the outcome—beyond the measured covariates—to fully explain away the observed association or to shift the confidence limit to the null.

## 3 Results

Approximately 17% of the sample reported having had a transformative RSE during adolescence. As a naturally occurring event, the timing of its first occurrence varied across individuals. The average age at which this experience took place was 18.77 years (SD = 2.34).

### 3.1 Antecedents of Transformative RSE

Table 1 summarizes the T0 characteristics of individuals who did and did not report a transformative RSE at T1. Those who reported such an experience were, on average, younger at T0, more likely to be female, and more likely to identify as Black rather than White.

Compared to those who did not report a transformative RSE at T1, individuals who did were characterized by several distinct features at T0. First, they tended to come from less advantaged financial backgrounds during adolescence, as indicated by lower household income quintiles and a higher likelihood of receiving welfare. Second, negative child‐parent dynamics were more prevalent in this group, including the absence of a parent, greater exposure to parental maltreatment, and higher levels of parental control. Third, they also tended to perform worse academically, with lower scores on the Peabody Picture Vocabulary Test (PPVT) and higher levels of delinquency. Fourth, both parental and personal health indicators were comparatively worse: their parents were more likely to have obesity, and they themselves reported more depressive symptoms, lower perceived life expectations (e.g., estimated chances of living to age 35), more somatic symptoms, and higher rates of suicidal ideation.

Finally, individuals who reported a transformative RSE were markedly more engaged in religious practice. Their mothers reported more frequent religious service attendance, and they themselves attended services more regularly and prayed more frequently. They also had sociodemographic characteristics typically associated with higher religiosity, such as residing in the American South, a region known for its strong religious culture (e.g., the Bible Belt). Additionally, this group was less likely to have a smoker in their household and reported lower levels of cigarette use—patterns often associated with religious discipline (Hussain et al. 2019).

### 3.2 Outcomes of Transformative RSE

The above descriptive results indicate that reporting a transformative RSE may be preceded by several factors indicative of an adverse adolescent environment, challenged parental and personal health, and heightened religious involvement. To account for these factors and reduce the risk of confounding or spurious associations (e.g., T2 depressive symptoms may be related to T0 depression, which may itself be associated with the reporting of a transformative RSE), the primary analysis examined associations between T1 transformative RSE and a wide range of T2 outcomes while adjusting for T0 covariates.

Table 2 presents the results for these associations across physical, behavioural, mental, and social domains of health and wellbeing. Beginning with the most robust findings—those meeting the Bonferroni correction threshold of *p* < 0.0013—reporting transformative RSE at T1 was significantly associated with a greater likelihood of volunteering at T2, RR = 1.21 [95% Confidence Interval: 1.12, 1.30] (*p* < 0.001, *E*‐value = 1.71, *E*‐limit = 1.48). This result suggests that individuals who reported a transformative RSE had a 21% higher likelihood of volunteering, even after controlling for T0 characteristics. An unmeasured confounder would need to be associated with both the exposure and the outcome with a risk ratio of at least 1.71 (or 1.48), above and beyond the measured covariates, to fully explain away this association (or shift the confidence limit to the null).

Similarly, transformative RSE was associated with a higher likelihood of voting, RR = 1.18 [1.09, 1.27]. In addition to these indicators of social engagement, the experience was also associated with higher levels of loneliness, with a standardized regression coefficient of *β* = 0.13 [0.06, 0.19]. This suggests that reporting a transformative RSE was linked to a 0.13 standard deviation increase in loneliness at T2.

Less robust findings—potentially subject to inflated Type I error—suggested several negative health outcomes following the experience. These included lower self‐rated health (*β* = −0.08 [−0.14, −0.01], *p* = 0.035), more depressive symptoms (*β* = 0.06 [0.00, 0.12], *p* = 0.044), higher likelihood of a PTSD diagnosis (OR = 1.54 [1.09, 2.19], *p* = 0.015), functional limitation (OR = 1.32 [1.04, 1.68], *p* = 0.022), diabetes (OR = 1.50 [1.03, 2.19], *p* = 0.035), and prescription drug misuse (RR = 1.15 [1.01, 1.31], *p* = 0.041). While some effect sizes were small, it is important to note that these outcomes were assessed at a relatively healthy stage of life, when such health problems are generally less prevalent. Notably, transformative RSE showed little evidence of association with most physical and behavioural health outcomes.

### 3.3 Sensitivity Analyses

The primary analysis controlled for a wide array of T0 covariates, which may have been overly conservative. To evaluate the influence of covariate adjustment on the findings, we reran the models using a reduced set of conventional sociodemographic covariates only (i.e., age, sex, race/ethnicity, nativity status, geographic region, family structure, household income, and parental education). Results, summarized in Supporting Information S1: Table S1, were strikingly similar: loneliness, voting, and volunteering remained robust outcomes (*p*s < 0.001). In addition to the previously identified negative outcomes, anxiety diagnosis and high cholesterol also emerged as statistically significant.

To address potential concerns about confounding due to the long lag between T0 covariates and the T1 exposure, we ran additional models adjusting for comparable contemporaneous variables measured at T1 (e.g., personal income, religious service attendance, physical inactivity, depressive symptoms, and voting; see notes of Supporting Information S1: Table S1). In these models, volunteering remained a robust outcome (RR = 1.14 [1.06, 1.24], *p* < 0.001), and loneliness, voting, and PTSD diagnosis also reached statistical significance (*p*s < 0.01). However, the previously observed negative physical and behavioural health outcomes were no longer statistically significant. Finally, we conducted a complete‐case analysis to compare with results from the imputed data. Due to reduced sample size and potential biases in error estimates, many effects no longer reached statistical significance. Nonetheless, volunteering remained a robust outcome (RR = 1.15 [1.05, 1.27], *p* < 0.01), along with loneliness (*β* = 0.10 [0.01, 0.18], *p* < 0.05). These results are presented in Supporting Information S1: Table S2.

Taken together, the findings indicate a robust and consistent association between reporting a transformative RSE and increased likelihood of volunteering. Less robust, but still noteworthy, associations were observed for loneliness, PTSD diagnosis, and voting.

## 4 Discussion

The current study assessed the longitudinal associations between reporting transformative RSE and a broad range of subsequent physical, mental, behavioural, and social health and wellbeing outcomes assessed approximately 7 years later, using a large, nationally representative sample of individuals transitioning from late adolescence to early adulthood. Two primary findings emerged that support our hypotheses. First, transformative RSE may occur along a pathway of psychological vulnerability, being preceded by stress‐inducing early life experiences and followed by poorer mental health. Second, as a potential contributing factor for connectedness, transformative RSE was associated with increased engagement in socially oriented behaviours.

Specifically, individuals who reported a transformative RSE at T1 were more likely to come from adverse and stressful childhood backgrounds, including lower household financial status, negative parent–child dynamics (e.g., maltreatment), poorer parental and personal health (e.g., elevated depressive symptoms), and, possibly as a consequence, lower academic performance. These individuals also showed markedly greater engagement in religious practices.

Following the occurrence of transformative RSE, respondents were more likely to report mental health difficulties and social distress—specifically, elevated loneliness and higher rates of PTSD diagnosis—as well as increased social engagement, including higher likelihood of volunteering and voting. These associations were consistent across three analytic strategies. The primary analysis adjusted for range of T0 characteristics, encompassing sociodemographic and family background, parental health and relationship quality, psychosocial and academic factors, physical health and behaviours, and religious/spiritual practice—many of which were correlated with the likelihood of reporting transformative RSE. A liberal model adjusted only for T0 demographic variables, while a conservative model adjusted for more proximal covariates measured at T1, including prior values of most outcomes.

It is noteworthy that in the conservative model, some of the health‐related associations observed in the primary analysis (e.g., higher rates of diabetes and functional limitations, poorer self‐rated health, and greater depressive symptoms) were no longer statistically significant after adjusting for covariates assessed at T1. This raises the possibility that adjustments based solely on more distal T0 variables may not adequately account for later mediating processes or be sufficient to rule out the possibility of reverse causality. However, the findings from the conservative model should also be interpreted with some caution, as the contemporaneous health and well‐being variables were assessed months or even years after the retrospectively reported transformative RSE. The attenuation of associations in the conservative model may reflect potential mediation or suppression effects from T1 health and wellbeing covariates, which could obscure the true longitudinal impact of transformative RSE.

### 4.1 Mental Health Vulnerability

The finding that transformative RSE are both preceded and followed by mental health and stress‐related factors aligns with the theoretical view that RSE, with its self‐weakening effect, may serve as a link within the broader chain of psychological vulnerability. Early life stressors may not only contribute to the development of conditions such as PTSD but also set the stage for transformative RSE, which may become an influencing factor in the trajectory of mental health vulnerability.

A critical element in understanding this covariate role is the influence of religious practice, which emerged as a strong correlate of reporting transformative RSE. This is not unexpected, as religious frameworks often provide both the conditions conducive to RSE and the interpretive structures necessary to make sense of such experiences. Indeed, religious practice was found to be the strongest predictor of religious or spiritual change in the 2004 U.S. General Social Survey. The demographic patterns observed in our study—namely, higher prevalence of transformative RSE among Black individuals and those living in the Southern United States—are consistent with those in the General Social Survey. These patterns were interpreted by Smith (2006) as reflections of regional and racial religious differences: ‘The higher level of spiritual/religious changes among blacks than among others and the low levels in New England and the high levels in the South largely reflect the religious differences’ (p. 293).

In addition, socioeconomic factors may play an important role. For example, evangelical religious affiliation is negatively associated with socioeconomic status in the United States. Members of the Southern Baptist Convention—the largest evangelical denomination—tend to have lower household incomes compared to members of other major religious groups (Pew Research Centre 2016). Combining these strands of evidence, perhaps the observed link between transformative RSE and household adversity (e.g., lower income) reflects the socioeconomic characteristics of certain evangelical populations concentrated in the American South.

From an influencer perspective, transformative RSE may have psychological consequences through the mechanism of failed or incomplete meaning‐making. Such experiences, particularly when highly intense or anomalous, can be difficult to integrate into one's existing worldview. Failure to do so may result in feelings of isolation and a heightened need for external validation. On one hand, some transformative experiences may be overwhelmingly positive, rendering ordinary life dull or even meaningless by contrast. Reflections from long‐term psychedelic users offer corroborative insights: ‘Once you have known the joy of becoming light, of dissolving into the crystalline body of the Divine, life on Earth can begin to feel dried up’ (Bache 2019, pp. 300–301). Transcendence, in this view, demands a reciprocal grounding in immanence—a capacity to remain embedded in the mundane aspects of life. On the other hand, some transformative RSE may be too bizarre or even distressing to assimilate, especially when they occur in individuals lacking a stable interpretive framework—such as adolescents early in their developmental trajectory in our study. Without sufficient life experience to contextualize these events, they may be appraised as negative and detrimental to mental health (Maraldi et al. 2024). The dual nature of transformative RSE has long been recognized. As Scottish psychiatrist Laing (1967) noted, ‘Madness need not be all breakdown. It may also be break‐through. It is potential liberation and renewal as well as enslavement and existential death’ (p. 110).

### 4.2 Opportunity for Connection

These findings should not be misinterpreted as pathologizing transformative RSE. Rather, their adaptive or maladaptive quality often depends on the surrounding cultural and interpretive context. Psychosis and RSE are not always distinguishable in form or content; both may arise in response to stress or existential crisis. What differs is the meaning ascribed to the experience. When interpreted as insightful and mobilized to resolve underlying distress, such experiences are often self‐limiting and non‐pathological (Dein 2010). Even among individuals with psychiatric diagnoses, reducing RSE to mere hallucinations or delusions strips them of their subjective and potentially therapeutic meaning. For example, in a qualitative study of 35 semi‐structured interviews with individuals in recovery from bipolar disorder in the Netherlands, participants described transformative RSE—including experiences of divine presence or unity—during manic episodes. Most interpreted these episodes as both spiritually authentic and intricately linked to their psychiatric condition (Ouwehand et al. 2018).

Importantly, for some individuals, the heightened sense of vulnerability and the need for connection following RSE appeared to translate into greater engagement in social life. One of the most consistent findings was an increased rate of volunteering. The co‐occurrence of loneliness and prosocial behaviour suggests a quest for connection, meaning, and belonging. Volunteering is well‐documented as a beneficial activity, associated with increased social contact, emotional support, and reduced mortality (Nakamura et al. 2025). A recent meta‐analysis of 21 studies across 191,652 participants found that volunteering may be a promising avenue for reducing loneliness (Akhter‐Khan et al. 2023). Moreover, two other meta‐analyses found a 22%–24% reduction in all‐cause mortality among volunteers compared to non‐volunteers, even after adjusting for potential sociodemographic and health‐related confounders (Jenkinson et al. 2013; Okun et al. 2013). That transformative RSE was robustly associated with increased volunteering strengthens the case for its double‐edged nature—by weakening or destabilizing the self, RSE may paradoxically open the door to greater unity and purpose beyond the self through connection and service to others.

This duality—vulnerability and connection—has important implications for the integration of RSE, especially in the context of psychedelic‐assisted therapies. While growing evidence supports the safety and therapeutic potential of these treatments (e.g., Griffiths et al. 2016), adverse effects must not be overlooked. The relative rarity of severe outcomes in existing clinical trials may be due to short‐term follow‐ups. A recent review of 214 studies involving 3504 participants found the longest follow‐up duration was just 60 weeks (Hinkle et al. 2024). In contrast, our study identified mental health vulnerabilities emerging seven or more years after the reported occurrence of transformative RSE. This suggests that while such experiences can yield lasting social benefits, their psychological risks may unfold slowly and remain undetected in shorter‐term evaluations. Therefore, efforts to integrate RSE—whether spontaneously occurring or pharmacologically induced—may benefit from long‐term support and attention to existential vulnerability as part of the therapeutic process.

### 4.3 Limitations, Implications and Conclusion

This study has several limitations inherent to the use of secondary data. First, our analyses were constrained by the available variables in the dataset. For example, we were unable to assess the effects of transformative RSE on meaning and purpose—a widely reported benefit in both qualitative research (Garcia‐Romeu et al. 2015) and clinical studies (Griffiths et al. 2006). In addition, the transformative RSE item was based on a single‐question format, which—as discussed in our introduction—may encompass a wide range of experiences depending on individual interpretation. Future studies would benefit from examining more specific subtypes of transformative RSE using multidimensional or phenomenologically grounded measures.

Second, self‐reported transformative RSE does not necessarily equate to an objectively verifiable event. Without examining the content of the experience, we cannot determine whether what is described truly qualifies as transformative. It is possible that vulnerabilities present at T0 may predispose individuals to interpret certain experiences as transformative RSE, or may make it more difficult for them to derive meaning from such experiences due to the ways these vulnerabilities shape their perceptions. Nonetheless, the belief that one has undergone such an experience often plays a crucial role in its perceived impact and significance. Studies incorporating qualitative or mixed‐methods data might provide valuable insight into the subjective meaning of transformative RSE in specific context (e.g., Chen and Guo 2025).

Third, our sample comprised individuals in late adolescence to early adulthood, a developmental stage characterized by heightened psychological and social transitions. Consequently, the findings may not generalize to other age groups. Many of the physical health variables analysed may be more salient for older populations, and the well‐documented benefits of volunteering have primarily been established among older adults. Thus, the implications of transformative RSE on younger individuals' health and social functioning may differ and warrant further exploration.

Fourth, although we employed a longitudinal, prospective design and adjusted for a wide range of potential confounders, this study is not intended to establish causality. As argued throughout, transformative RSE may be best situated along a broader trajectory of vulnerability rather than functioning as a direct trigger of change. While the effect of RSE on volunteering was statistically robust, it was modest in size; in addition, these are population average effects, so some people may have the positive effects of transformative RSE while some others have negative effects. Therefore, the full range of its potential benefits remains to be investigated in future research.

The robust and consistent association between reporting transformative RSE and increased likelihood of volunteering and voting suggests that such experiences may serve as a catalyst for prosocial behaviours and civic engagement at a population level, offering valuable leverage points for mental health professionals, educators, and spiritual caregivers. For practitioners, recognizing and integrating discussions around transformative RSE could foster greater community engagement and resilience in clients or students.

In conclusion, this study demonstrated the double‐edged nature of transformative RSE using a large, longitudinal dataset. Such experiences appear to destabilize the self, exposing individuals to psychological vulnerability, while simultaneously opening up possibilities for greater connection through prosocial engagement. Although the content and metaphysical assumptions underlying these experiences vary widely, they consistently point to a central theme long recognized by James (1902): ‘The only thing [religious experience] unequivocally testifies to is that we can experience union with something larger than ourselves and in that union find our greatest peace’ (p. 518).

## Conflicts of Interest

The authors declare no conflicts of interest.

## Supporting information


Supporting Information S1

## Data Availability

Data sharing not applicable to this article as no datasets were generated or analysed during the current study.
