# Supplementary material for: Between Vulnerability and Connection: Longitudinal Evidence on the Impact of Transformative Religious/Spiritual Experiences
Source: Stress Health. 2025 Oct 3;41(5):e70110. doi: 10.1002/smi.70110 (PMC12495122; doi:10.1002/smi.70110)
Supplement: Supplementary file 1 — Supporting Information S1 [file SMI-41-e70110-s001.docx]

**Supplementary Online Content**

# Between Vulnerability and Connection: Longitudinal Evidence on the Impact of Transformative Religious/Spiritual Experiences

**Supplementary Text S1.** Assessment of Outcomes

**Supplementary Table S1.** Associations of T1 (2001-2002) reporting a transformative R/S experience with subsequent health and wellbeing at T2 (2008), controlling for T0 (1994-1995) conventional covariates or T1 contemporaneous covariates (*n* = 10,529)

**Supplementary Table S2.** Associations of T1 reporting a transformative R/S experience with subsequent health and wellbeing in adulthood at T2, controlling for T0 characteristics – complete-case analysis (*n* ranged from 5,013 to 5,391)

# Supplementary Text S1 Assessment of Outcomes

## Physical Health

**Diagnosed Physical Health Conditions.** Participants self-reported whether they had ever been told by a doctor, nurse, or other health care provider that they had: a) cancer, b) high cholesterol, c) high blood pressure or hypertension (for women, when not pregnant), d) diabetes (for women, when not pregnant), e) asthma, or f) migraine headaches.

**Allostatic Load.** We created an allostatic load score for participants using biomarkers of stress, use of biomarker-regulating medications, and self-reports of health conditions related to the biomarkers. Add Health interviewers collected blood pressure, obtained blood samples for cholesterol and glucose from participants, and inventoried prescription medications during the in-home interviews. Following the approach used by Richardson, Goodwin, and Hummer (2021), we determined the high-risk threshold for each biomarker and gave one point per biomarker value beyond that threshold. The high-risk threshold was above the 75th percentile of all values in the sample for diastolic blood pressure (DBP), systolic blood pressure (SBP), pulse rate, waist circumference, hemoglobin A1c (HbA1c), and C-reactive protein (CRP); above the 80th percentile for triglycerides and total cholesterol; and below the 20th percentile for high-density lipoprotein (HDL) cholesterol. Among respondents who were not identified as high risk on a particular biomarker based on the sample-derived cutoffs, a point was given for use of a biomarker-regulating medication or a self-reported diagnosis of a condition related to a biomarker. In particular, for participants who were taking medication to manage hypertension, diabetes, hyperlipidemia, or inflammation, and/or those who reported receiving a diagnosis for hypertension, diabetes, or hyperlipidemia, a point was given for SBP, DBP, HbA1c, total cholesterol, triglycerides, HDL, or CRP, respectively. To calculate the total allostatic load score, the points based on biomarker levels, medication use, and self-reported conditions were summed, yielding a single score that ranged from 0 to 9 for each participant.

**Overweight/Obesity.** Add Health interviewers collected measures of participant weight and height and constructed categories of body mass index (BMI, kg/m2) derived from these measurements: underweight (<18.5), normal (18.5-<25), overweight (25-<30) or obese I (30-<35), obese II (35-<40), or obese III (40+). Participants with BMI categories of overweight or obese (I, II, or III) were assigned a code of 1 (*overweight/obese*) while those in the underweight or normal categories were coded as 0 (*not overweight/obese*).

**Functional Limitations.** Function limitations were assessed using an item asking participants about the extent to which their health limits their moderate activities (e.g., moving a table, pushing a vacuum cleaner, etc.). Responses indicating any limitations (i.e., “limited a little” or “limited a lot”) were coded as 1 (*functional limitations*) versus none (i.e., “not limited”) were coded as 0 (*no functional limitations*).

**Cognition.** Add Health interviewers performed three memory-ability tasks on participants: immediate word recall, delayed word recall, and digits backward recall. The word recall tasks involved interviewers reading a list of words and asking participants to relay back as many as they could remember in 90 seconds. The digit recall task used a set of numbers that participants were asked to recall back in reverse order, with interviewers repeating longer series of numbers each time up to seven times. The scores for each task were determined by the number of correct words [or numbers]. We standardized the scores on all three measures to have means of zero and standard deviations of one, and then summed the three measures together and re-standardized (Stebbins et al., 2022).

**Self-Rated Health.** Participants were asked “In general, how is your health?” with response categories including excellent, very good, good, fair, and poor. The measure was coded such that higher scores reflected better self-rated health.

## Behavioral Health

**Sleep Disturbance.** Sleep disturbance was assessed using two items asking participants over the past four weeks, “how often did you have trouble falling asleep?” and “how often did you have trouble staying asleep through the night?” Response options for both items included “never,” “less than once a week,” “1 or 2 times a week,” “3 or 4 times a week,” and “5 or more times a week.” We combined the two items for trouble falling and staying asleep to create a dichotomous indicator such that “had trouble falling (or staying) asleep 3 or 4 times a week or more in the past four weeks” was coded as 1 (*sleep disturbance*), and “had trouble falling (or staying) asleep 1 or 2 times a week or less” was coded as 0 (*no sleep disturbance)* (Fricke and Sironi, 2017).

**Physical Inactivity.** Participants were asked about their frequency of activity sessions across a range of different physical activities (e.g., bicycling, running, golfing, roller blading, walking for exercise) in the past seven days. Response options ranged from 0 (Not at all) to 7 (7 or more times), and the binary variable was created to by assigning 1 for no participation in the past week in any of the physical activities listed (*physical inactivity*) and 0 for any physical activity in the past week (*not physically inactive*).

**Cigarette Smoking.** Cigarette smoking was assessed by two items asking participants: 1) “Have you ever smoked cigarettes regularly--that is, at least one cigarette every day for 30 days?” and 2) During the past 30 days, on how many days did you smoke cigarettes?” Participants who reported they had ever smoked cigarettes regularly and smoked cigarettes on at least one of the past 30 days were coded 1 (*cigarette smoking*) and 0 for with never smoking regularly or not smoking at all in the past month (*no cigarette smoking*).

**Binge Drinking.** Participants were asked about their alcohol consumption during the past 12 months, including on how many days they drank 5 or more drinks in a row (if male; 4 or more if female). Response options were: ‘none,’ ‘1 or 2 days,’ ‘once a month or less (3 to 12 days in the past 12 months),’ ‘2 or 3 days a month,’ ‘1 or 2 days a week,’ ‘3 to 5 days a week,’ and ‘every day or almost every day.’ Those who reported binge drinking at least 1 or 2 days a week were assigned a code of 1 (*weekly binge drinking*), whereas those who reported binge drinking less than once a week or never were assigned a code of 0 (*no weekly binge drinking*) (Patrick and Schulenberg 2014).

**Marijuana Use.** Marijuana use was assessed using an item that asked participants, “During the past 30 days, how many times did you use marijuana?” Responses indicating any marijuana use in the past month were coded as 1 (*marijuana use*) and responses of zero times or legitimate skip, which was assigned for never having used marijuana, were coded as 0 (*no marijuana use*).

**Prescription Drug Misuse.** Participants were asked whether they had used prescription drugs in ways they were not intended, including taking prescription drugs “that were not prescribed for [them],” “in larger amounts than prescribed, more often than prescribed, for longer periods than prescribed,” or “only for the feeling or experience they caused.” Affirmative answers were coded as 1 (*prescription drug misuse)* and negative responses were coded as 0 (*no prescription drug misuse*).

**Illicit Drug Use.** Participants reported whether they had ever used any of the following drugs: a) steroids, b) cocaine, c) crystal meth, d) other. Any response of ‘yes’ was coded as 1 (*illicit drug use*) and responses of ‘no’ across all items were coded as 0 (*no illicit drug use*).

**History of Sexually Transmitted Infections.** Participants self-reported whether in the past 12 months they had been told by a doctor, nurse, or other health professional that they had any of the following sexually transmitted infections: a) chlamydia, b) gonorrhea, c) trichomoniasis, d) syphilis, e) genital herpes, f) genital warts, g) hepatitis B (HBV), h) human papilloma virus (HPV), i) pelvic inflammatory disease (PID), j) cervicitis or mucopurulent cervicitis (MPC), k) urethritis, l) vaginitis, m) HIV infection or AIDS, or n) any other sexually transmitted disease. Reports of any diagnosis of a sexually transmitted infections (STIs) were coded as 1 (*history of STIs*), while reports of no diagnoses across all types of sexually transmitted infections were coded as 0 (*no history of STIs*).

**Preventative Health Care Use.** Preventative health care use was assessed using an item that asked participants about how long ago they last had a routine check-up. Response options were ‘within the past 3 months,’ ‘4 to 6 months ago,’ ‘7 to 9 months ago,’ ‘10 to 12 months ago,’ ‘longer than 1 year ago but less than 2 years ago,’ ‘2 years ago or longer,’ or ‘never.’ Responses indicating the most recent health care visits were within the past 12 months were coded 1 (*preventative health care use*) and health care visits more than 12 months in the past were coded as 0 (*no preventative health care use*).

## Mental Health and Wellbeing

**Depressive Symptoms***.* The measure for depressive symptoms consists of nine items that asked participants how often during the past seven days they experienced the following feelings: 1) bothered by things, 2) blue, 3) as good as others, 4) trouble focusing, 5) depressed, 6) tired, 7) enjoying life, 8) sad, and 9) disliked by others. Response options were ‘never or rarely,’ ‘sometimes,’ ‘a lot of the time,’ and ‘most of the time or all of the time.’ After reverse coding positively valanced items (items 3 and 7), all items were averaged together to create a composite score, with higher scores indicating higher depressive symptoms (α = 0.81, range: 1 to 4).

**Diagnosed Mental Health Conditions.** Participants self-reported whether they had ever been told by a doctor, nurse, or other health care provider that they have or had any of the following mental health conditions: a) depression, b) anxiety or panic disorder, c) post-traumatic stress disorder or PTSD, d) attention problems or ADD or ADHD.

**Suicidal Ideation.** Suicidal ideation was assessed by an item asking participants: “During the past 12 months, have you ever seriously thought about committing suicide?” Affirmative responses were coded 1 (*suicidal ideation*) and negative responses were coded 0 (*no suicidal ideation*).

**Perceived Stress.** Perceived stress was measured with four items asking participants how often in the last 30 days they felt: 1) they were unable to control the important things in their life, 2) they were confident in their ability to handle their personal problems, 3) things were going their way, and 4) difficulties were piling up so high that they could not overcome them. Response options included ‘never,’ ‘almost never,’ ‘sometimes,’ ‘fairly often,’ and ‘very often.’ After reverse coding positively worded items (items 2 and 3), the four items were averaged together to create a composite score, with higher scores indicating higher perceived stress (α = 0.72, range: 1 to 5) (Lippert and Damaske, 2019).

**Job Satisfaction.** Participants were asked about their satisfaction with their current or most recent job: “How satisfied (are/were) you with this job, as a whole?” Response categories ranged from 1 (extremely satisfied) to 5 (extremely dissatisfied), and the measure was reverse coded such that higher scores reflected greater job satisfaction.

**Optimism***.* Optimism was assessed using four items that asked participants about their level of agreement with four statements about how they generally are now, not as they wish to be in the future: 1) “I'm always optimistic about my future,” 2) “I hardly ever expect things to go my way,” 3) “Overall, I expect more good things to happen to me than bad,” and 4) “I rarely count on good things happening to me.” Response options ranged from 1 (strongly agree) to 5 (strongly disagree). After coding each item so that higher scores reflected stronger agreement with the positively valanced statements (items 1 and 3) and stronger disagreement with the negatively worded statements (items 2 and 4), the four items were averaged together to create a single composite measure with higher scores reflecting higher optimism (α = 0.66, range: 1 to 5) (Fletcher, 2020).

**Sense of Control***.* Sense of control was assessed using five items that asked participants about their level of agreement with the following statements: 1) “There is little I can do to change the important things in my life,” 2) “Other people determine most of what I can and cannot do,” 3) “There are many things that interfere with what I want to do,” 4) “There is really no way I can solve the problems I have,” and 5) “I am not interested in other people's problems.” Response options ranged from 1 (strongly agree) to 5 (strongly disagree). All items were reverse coded and then averaged together to create a composite measure, with higher scores indicating higher sense of control (α = 0.76, range:1 to 5) (McFarland et al., 2016).

## Social Wellbeing and Engagement

**Loneliness***.* Loneliness was assessed with an item that asked participants: “How often do you feel isolated from others?” Response options included ‘never,’ ‘rarely,’ ‘sometimes,’ and ‘often,’ with higher scores indicating higher loneliness.

**Romantic Relationship Quality.** We used seven items that asked participants about the extent to which they agreed or disagreed with the following statements about their relationship with their current or most recent partner: 1) “We [enjoy/enjoyed] doing even ordinary, day-to-day things together,” 2) “I [am/was] satisfied with the way we handle our problems and disagreements,” 3) “I [am/was] satisfied with the way we handle family finances,” 4) “My partner [listens/listened] to me when I need someone to talk to,” 5) “My partner [expresses/expressed] love and affection to me,” 6) “I [am/was] satisfied with our sex life,” and 7) “I [trust/trusted] my partner to be faithful to me.” Response options ranged from 1 (strongly agree) to 5 (strongly disagree). After reverse coding all items such that higher scores indicated stronger agreement with the statements, they were averaged together to create a composite measure, with higher scores indicating greater romantic relationship quality (α = 0.89, range: 1 to 5) (Washington, 2020).

**Parenting Satisfaction.** Parenting satisfaction was assessed using four items that asked participants who reported that they had children about their level of agreement with the following statements: 1) “I am happy in my role as a parent,” 2) “I feel close to my child(ren),” 3) “The major source of stress in my life is my child(ren),” and 4) “I feel overwhelmed by the responsibility of being a parent.” Response options ranged from 1 (strongly agree) to 5 (strongly disagree). After coding each item so that higher scores reflected stronger agreement with the positively valanced statements (items 1 and 2) and stronger disagreement with the negatively worded statements (items 3 and 4), the four items were averaged together to create a composite measure, with higher scores indicating higher parenting satisfaction (α = 0.58, range: 1 to 5) (Beaver et al., 2014).

**Relationship Quality with a Parent.** Participants were asked about their relationships with their parental figures, including their level of closeness with their mother and/or father: “How close do you feel to your [mother/father figure]?” Response options included ‘not at all close,’ ‘not very close,’ ‘somewhat close,’ ‘quite close,’ and ‘very close.’ We used the maximum score between the two assessments (for closeness with mother and/or father), with higher scores indicating greater relationship quality with a parent.

**Voting***.* Participants were asked about the following question about their voting behavior: “How often do you usually vote in local or statewide elections?” Response options included ‘never,’ ‘sometimes,’ ‘often,’ or ‘always.’ We created a binary measure with responses of voting ‘often’ or ‘always’ coded as 1 (*voting*) and responses of ‘sometimes’ or ‘never’ coded as 0 (*no voting*).

**Volunteering***.* Volunteering was assessed using an item that asked participants, “In the past 12 months, how many hours did you spend on volunteer or community service work?” Response categories included ‘0 hours,’ ‘1 to 19 hours,’ ‘20 to 39 hours,’ ‘40 to 79 hours,’ ‘80 to 159 hours,’ and ‘160 hours or more.’ We created a binary measure to represent volunteering in the past year by coding participants reporting any hours (i.e., 1 hour or more) of volunteering or community service work as 1 (*any volunteering*) and those reporting no hours of volunteering as 0 (*no volunteering*).

## References

Beaver, K. M., da Silva Costa, C., Poersch, A. P., Freddi, M. C., Stelmach, M. C., Connolly, E. J., & Schwartz, J. A. (2014). Psychopathic personality traits and their influence on parenting quality: Results from a nationally representative sample of Americans. *Psychiatric Quarterly*, *85*, 497-511.

Fletcher, J. (2020). Assessing the importance of childhood context in the development of hope and optimism. *Journal of Happiness Studies*, *21*, 2419-2427.

Fricke, J., & Sironi, M. (2017). Dimensions of sexual orientation and sleep disturbance among young adults. *Preventive Medicine Reports*, *8*, 18-24.

Lippert, A. M., & Damaske, S. (2019). Finding jobs, forming families, and stressing out? Work, family, and stress among young adult women in the United States. *Social Forces*, *98*(2), 885-914.

McFarland, M. J., Wagner, B., & Marklin, S. (2016). College education and sense of control: A twin-discordant design. *Socius*, *2*, 2378023116656011.

Richardson, L. J., Goodwin, A. N., & Hummer, R. A. (2021). Social status differences in allostatic load among young adults in the United States. *SSM-Population Health*, *15*, 100771.

Stebbins, R. C., Yang, Y. C., Reason, M., Aiello, A. E., Belsky, D. W., Harris, K. M., & Plassman, B. L. (2022). Occupational cognitive stimulation, socioeconomic status, and cognitive functioning in young adulthood. *SSM-Population Health*, *17*, 101024.

Washington, C. (2021). Romantic relationship quality of youth with two biological parents and stepfathers. *Journal of Family Issues*, *42*(6), 1333-1353.

# Supplementary Table S1

Associations of T1 (2001-2002) reporting a transformative R/S experience with subsequent health and wellbeing at T2 (2008), controlling for T0 (1994-1995) conventional covariates or T1 contemporaneous covariates (*n* = 10,529)

|  | Conventionally adjusted models ^a^ | | Contemporaneously adjusted models ^b^ | |
| --- | --- | --- | --- | --- |
| Outcomes assessed at T2 | β [95% CI] | RR/OR [95% CI] | β [95% CI] | RR/OR [95% CI] |
| **Physical health** |  |  |  |  |
| Number of diagnosed conditions | 0.05 [-0.02, 0.12] |  | -0.01 [-0.07, 0.06] |  |
| Cancer |  | 1.04 [0.59, 1.84] |  | 0.89 [0.46, 1.73] |
| High cholesterol |  | 1.32 [1.02, 1.72]* |  | 1.20 [0.90, 1.59] |
| Hypertension |  | 1.02 [0.85, 1.23] |  | 0.99 [0.81, 1.21] |
| Diabetes |  | 1.67 [1.16, 2.42]** |  | 1.50 [0.99, 2.28] |
| Asthma |  | 1.10 [0.93, 1.29] |  | 0.96 [0.84, 1.10] |
| Migraines |  | 0.92 [0.77, 1.10] |  | 0.87 [0.72, 1.05] |
| Allostatic load | 0.04 [-0.03, 0.11] |  | 0.02 [-0.04, 0.08] |  |
| Overweight/obesity |  | 1.02 [0.96, 1.07] |  | 1.00 [0.96, 1.05] |
| Functional limitations |  | 1.32 [1.05, 1.68]* |  | 1.22 [0.94, 1.58] |
| Cognition | 0.06 [-0.01, 0.12] |  | 0.02 [-0.05, 0.09] |  |
| Self-rated health | -0.09 [-0.16, -0.01]* |  | -0.05 [-0.12, 0.02] |  |
| **Behavioral health** |  |  |  |  |
| Sleep disturbance |  | 1.04 [0.97, 1.11] |  | 1.02 [0.95, 1.09] |
| Physical inactivity |  | 0.96 [0.80, 1.14] |  | 0.97 [0.81, 1.17] |
| Cigarette smoking |  | 0.99 [0.89, 1.10] |  | 1.01 [0.92, 1.10] |
| Binge drinking |  | 0.91 [0.74, 1.11] |  | 0.97 [0.80, 1.18] |
| Marijuana use |  | 1.11 [0.94, 1.31] |  | 1.12 [0.96, 1.30] |
| Prescription drug misuse |  | 1.19 [1.04, 1.37]* |  | 1.10 [0.98, 1.24] |
| Illicit drug use |  | 1.12 [1.00, 1.26] |  | 1.09 [0.99, 1.19] |
| History of STIs |  | 1.00 [0.82, 1.22] |  | 0.95 [0.77, 1.17] |
| Preventative health care use |  | 0.98 [0.93, 1.03] |  | 0.96 [0.91, 1.02] |
| **Mental health and wellbeing** |  |  |  |  |
| Depressive symptoms | 0.09 [0.03, 0.16]** |  | 0.03 [-0.03, 0.09] |  |
| Depression diagnosis |  | 1.16 [0.98, 1.37] |  | 0.95 [0.81, 1.10] |
| Anxiety diagnosis |  | 1.24 [1.06, 1.45]** |  | 1.14 [0.96, 1.34] |
| PTSD diagnosis |  | 1.69 [1.18, 2.41]** |  | 1.73 [1.15, 2.60]** |
| ADD/ADHD diagnosis |  | 1.08 [0.78, 1.49] |  | 0.86 [0.59, 1.25] |
| Suicidal ideation |  | 1.39 [1.07, 1.81]* |  | 1.18 [0.88, 1.57] |
| Perceived stress | 0.02 [-0.04, 0.09] |  | -0.01 [-0.07, 0.06] |  |
| Happiness | -0.07 [-0.15, 0.01] |  | -0.04 [-0.11, 0.04] |  |
| Job satisfaction | -0.00 [-0.08, 0.08] |  | 0.00 [-0.07, 0.08] |  |
| Optimism | 0.05 [-0.02, 0.13] |  | 0.07 [-0.01, 0.14] |  |
| Sense of control | -0.02 [-0.10, 0.05] |  | -0.00 [-0.07, 0.07] |  |
| **Social wellbeing and engagement** |  |  |  |  |
| Loneliness | 0.16 [0.10, 0.22]*** |  | 0.10 [0.04, 0.17]** |  |
| Romantic relationship quality | -0.04 [-0.10, 0.03] |  | -0.03 [-0.10, 0.03] |  |
| Satisfaction with parenting ^ | -0.05 [-0.15, 0.04] |  | -0.06 [-0.15, 0.03] |  |
| Relationship quality with parent | 0.01 [-0.06, 0.09] |  | 0.03 [-0.04, 0.10] |  |
| Voting |  | 1.22 [1.13, 1.32]*** |  | 1.12 [1.03, 1.21]** |
| Volunteering |  | 1.28 [1.18, 1.39]*** |  | 1.14 [1.06, 1.24]*** |

Note. RR, risk ratio; OR, odds ratio; CI, confidence interval. **p* < .05, ***p* < .01, ****p* < .001

^a^ All models controlled for age, sex, race/ethnicity, nativity status, geographic region, family structure, household income, and parental education assessed at T0.

^b^ All models controlled for age, sex, race/ethnicity, and nativity status assessed at T0, and sexual orientation, marital status, parenthood status, high school equivalency, currently enrolled in school, employment status, health insurance status, personal income, home ownership status, welfare receipt, religious service attendance, overweight/obesity, functional limitations, depression diagnosis, depressive symptoms, suicidal ideation, life satisfaction, physical inactivity, cigarette smoking, binge drinking, marijuana use, illicit drug use, history of STIs, preventative health care use, relationship quality with a parent, and voting at T1.

^ Analysis for this outcome was restricted to participants who reported having at least one child at T2 (*n* = 5,172).

# Supplementary Table S2

Associations of T1 reporting a transformative R/S experience with subsequent health and wellbeing in adulthood at T2, controlling for T0 characteristics – complete-case analysis (*n* ranged from 5,013 to 5,391)

| Outcomes assessed at T2 | β [95% CI] | RR/OR [95% CI] |
| --- | --- | --- |
| **Physical health** |  |  |
| Number of diagnosed conditions | 0.01 [-0.10, 0.12] |  |
| Cancer |  | 1.11 [0.51, 2.41] |
| High cholesterol |  | 1.25 [0.91, 1.74] |
| Hypertension |  | 0.96 [0.70, 1.31] |
| Diabetes |  | 1.60 [0.91, 2.83] |
| Asthma |  | 0.96 [0.80, 1.15] |
| Migraines |  | 0.89 [0.71, 1.13] |
| Allostatic load | 0.01 [-0.08, 0.10] |  |
| Overweight/obesity |  | 1.02 [0.96, 1.09] |
| Functional limitations |  | 1.22 [0.86, 1.75] |
| Cognition | 0.04 [-0.05, 0.13] |  |
| Self-rated health | -0.05 [-0.13, 0.04] |  |
| **Behavioral health** |  |  |
| Sleep disturbance |  | 0.98 [0.89, 1.09] |
| Physical inactivity |  | 1.00 [0.78, 1.27] |
| Cigarette smoking |  | 1.03 [0.89, 1.20] |
| Binge drinking |  | 0.86 [0.67, 1.11] |
| Marijuana use |  | 1.12 [0.92, 1.37] |
| Prescription drug misuse |  | 1.04 [0.84, 1.28] |
| Illicit drug use |  | 1.13 [0.95, 1.36] |
| History of STIs |  | 0.95 [0.71, 1.28] |
| Preventative health care use |  | 0.94 [0.87, 1.02] |
| **Mental health and wellbeing** |  |  |
| Depressive symptoms | 0.05 [-0.03, 0.13] |  |
| Depression diagnosis |  | 1.14 [0.91, 1.42] |
| Anxiety diagnosis |  | 1.20 [0.97, 1.47] |
| PTSD diagnosis |  | 1.59 [0.91, 2.77] |
| ADD/ADHD diagnosis |  | 1.68 [0.90, 3.12] |
| Suicidal ideation |  | 1.30 [0.88, 1.92] |
| Perceived stress | 0.05 [-0.04, 0.13] |  |
| Happiness | -0.01 [-0.10, 0.08] |  |
| Job satisfaction | 0.00 [-0.09, 0.09] |  |
| Optimism | -0.02 [-0.11, 0.08] |  |
| Sense of control | -0.08 [-0.17, 0.02] |  |
| **Social wellbeing and engagement** |  |  |
| Loneliness | 0.10 [0.01, 0.18]* |  |
| Romantic relationship quality | -0.04 [-0.12, 0.05] |  |
| Satisfaction with parenting ^ | -0.15 [-0.29, -0.01]* |  |
| Relationship quality with parent | 0.05 [-0.04, 0.14] |  |
| Voting |  | 1.10 [1.00, 1.21] |
| Volunteering |  | 1.15 [1.05, 1.27]** |

Note. RR, risk ratio; OR, odds ratio; CI, confidence interval. *p < .05, **p < .01, ***p < .001. All models controlled for **sociodemographic and family background** (age, sex, race/ethnicity, nativity status, geographic region, family structure, number of siblings, household income, household welfare receipt, insurance status, mother age, mother race/ethnicity, parent nativity, parental education, mother employment status), **parental health and relationship** (mother health status, mother happiness, parent has a disability, parent has obesity, parent has alcoholism, smoker in household, childhood maltreatment by parents, parental control, relationship quality with a parent); **psychosocial and academic factors** (mental health condition diagnosis, depressive symptoms, happiness, self-esteem, life expectancy, romantic relationship status, has a learning disability, school connectedness, neighborhood social cohesion, PPVT, GPA, delinquency); **health status and behavior** (somatic symptoms, pubertal development, physical health condition diagnosis, overweight/obesity, functional limitations, self-rated health, suicidal ideation, sleep disturbance, physical inactivity, cigarette smoking, binge drinking, marijuana use, illicit drug use, history of STIs, preventative health care use); and **religious/spiritual practice** (mother religious service attendance, religious service attendance, frequency of prayer) assessed at T0. ^Analysis for this outcome was restricted to participants who reported having at least one child at T2 (*n* = 2,406).
